# A Comparative Assessment of Clinical Outcomes Using Cyanoacrylate Glue versus Subcuticular Sutures in Cosmetic Procedures at a Tertiary Care Center: Evaluation Based on the Southampton Scoring System

**DOI:** 10.7759/cureus.75484

**Published:** 2024-12-10

**Authors:** Kedara Harshitha, Subramani Balakrishnan, Evangeline P Christina, Alexander Mecheri Antony, Karpagam R Kannadasan

**Affiliations:** 1 General Surgery, Sree Balaji Medical College and Hospital, Chennai, IND; 2 Radiology, Saveetha Medical College and Hospital, Saveetha Institute of Medical and Technical Sciences (SIMATS) Saveetha University, Chennai, IND

**Keywords:** cosmetic procedures, cyanoacrylate glue, southampton scoring system, subcuticular sutures, wound healing

## Abstract

Introduction

Cosmetic surgery has advanced significantly, with wound closure techniques crucial for determining aesthetic and healing outcomes. Recently, cyanoacrylate glue and subcuticular sutures have gained attention for their unique benefits in cosmetic procedures. Cyanoacrylate glue, a non-invasive tissue adhesive, facilitates faster wound closure with minimal trauma, while subcuticular sutures offer durable, concealed closures, particularly suited for areas under mechanical stress. This study uses the Southampton scoring system to compare these techniques, evaluating clinical outcomes, complications, and patient satisfaction to guide optimal wound closure methods in cosmetic surgery.

Methods

This prospective observational study at a tertiary care center compared wound closure outcomes using cyanoacrylate glue and subcuticular sutures in 50 patients undergoing cosmetic procedures. Patients aged 18-65 with no wound healing disorders or chronic skin conditions were included, while those with active infections, on immunosuppressive therapy, or allergic to cyanoacrylate were excluded. Patients were evenly divided into two groups: one receiving cyanoacrylate glue and the other subcuticular sutures. The same surgical team performed all procedures. Wound healing was assessed using the Southampton scoring system on days 1, 7, and 30 postoperatively. Secondary outcomes included pain (visual analog scale), healing time, and complication rates. Patient satisfaction was evaluated at the 30-day follow-up. Statistical analysis was conducted using SPSS software, with significance set at p<0.05.

Results

The study evaluated 50 patients (split equally between cyanoacrylate glue and subcuticular sutures groups) undergoing cosmetic procedures, focusing on postoperative wound healing using the Southampton scoring system. On postoperative day 7, infection rates were significantly lower with cyanoacrylate glue (8% Grade 1a, 4% Grade 1c, and no Grade 2b) than with subcuticular sutures (16% Grade 1a, 12% Grade 1c, and 4% Grade 2b; p=0.000). Additionally, cyanoacrylate glue reduced operative time, with its non-invasive nature contributing to higher patient satisfaction scores. However, both techniques yielded comparable long-term cosmetic outcomes, with no significant differences in scar appearance after 30 days. Overall, each technique demonstrated unique strengths, influencing clinical outcomes and patient satisfaction.

Conclusion

The study highlights the respective benefits and limitations of cyanoacrylate glue and subcuticular sutures, offering insights to aid cosmetic surgeons in making informed decisions tailored to individual patient needs. These findings can ultimately improve short-term recovery and long-term patient satisfaction with cosmetic outcomes. The use of the Southampton scoring system facilitated a standardized evaluation, providing valuable data on the effectiveness of each wound closure method in various surgical contexts.

## Introduction

Cosmetic surgery has rapidly evolved over the years, with wound closure techniques playing a pivotal role in determining postoperative outcomes, particularly in terms of aesthetics and healing. In recent years, there has been significant interest in comparing various wound closure methods to enhance cosmetic results and patient satisfaction. Two prominent techniques that have gained attention in cosmetic procedures are cyanoacrylate glue and subcuticular sutures [1]. Each of these methods offers unique benefits and potential drawbacks, making it crucial to evaluate their effectiveness through a standardized approach, such as the Southampton scoring system.

Cyanoacrylate glue, a tissue adhesive, has been increasingly favored due to its non-invasive nature, ease of use, and ability to expedite wound closure. The adhesive creates a strong bond between skin edges without the need for traditional sutures, reducing needle trauma and patient discomfort [2]. Studies have suggested that cyanoacrylate glue may lead to faster healing times and improved cosmetic outcomes, especially in minor and medium-sized wounds. Moreover, its quick application and elimination of the need for suture removal make it an appealing option for patients and surgeons alike.

On the other hand, subcuticular sutures have been a long-standing method for closing wounds, particularly in cosmetic and plastic surgeries. This technique involves the placement of absorbable sutures just beneath the skin’s surface, offering a more concealed closure that reduces visible scarring. Subcuticular sutures are known for providing robust wound approximation and durability, which are essential in areas subjected to mechanical stress. However, this method may require more time for application and can sometimes result in increased discomfort during the healing process.

The Southampton scoring system is a valuable tool for objectively assessing postoperative wound healing. It evaluates key aspects of wound recovery, such as inflammation, infection, discharge, and the integrity of the wound edges. By using this system, clinicians can standardize their evaluation of cosmetic procedures and draw meaningful comparisons between different closure techniques [3]. In this study, the Southampton scoring system is employed to compare the clinical outcomes of cyanoacrylate glue and subcuticular sutures in cosmetic surgery patients.

The comparative analysis of cyanoacrylate glue and subcuticular sutures is not only pertinent for improving patient outcomes but also for optimizing surgical efficiency. As surgeons continuously seek methods that enhance both aesthetic results and patient comfort, understanding the nuances between these closure techniques becomes increasingly important [4]. This study focuses on clinical parameters such as cosmetic appearance, complication rates, and patient satisfaction to provide a comprehensive overview of the strengths and weaknesses of each method.

In conclusion, this study aims to shed light on the clinical performance of cyanoacrylate glue versus subcuticular sutures in cosmetic procedures performed at a tertiary care center. Through the Southampton scoring system, this study provides a structured and standardized assessment of wound healing and complications, ultimately guiding future surgical decisions for improved patient care [5]. The findings will offer insights into the most effective techniques for achieving optimal cosmetic outcomes while minimizing complications.

## Materials and methods

This study was conducted at a tertiary care center, focusing on patients undergoing various cosmetic procedures. The primary aim was to compare the clinical outcomes of wound closure techniques using cyanoacrylate glue and subcuticular sutures. Ethical approval was obtained from Sree Balaji Medical College and Hospital institutional human ethics committee (002/SBMCH/IHEC/2022/1866), and informed consent was acquired from all participants before the commencement of the study.

The research followed a prospective observational design, with 50 patients recruited over a 12-month period and equally divided into two groups. One group underwent wound closure using cyanoacrylate glue, while the other received subcuticular sutures. The study's inclusion criteria consisted of patients aged 18-65 years undergoing elective cosmetic procedures with no prior history of wound healing disorders or chronic skin conditions. Patients were excluded if they had active infections, were on immunosuppressive therapy, or had hypersensitivity to cyanoacrylate glue.

All cosmetic procedures were performed by the same surgical team to minimize variability between cases. In the cyanoacrylate glue group, the adhesive was applied to the wound edges immediately following surgery, whereas the subcuticular sutures group received standard absorbable sutures. Each procedure was carefully documented using standardized protocols to ensure consistency across all patients.

The primary outcome measure was the quality of wound healing, evaluated using the Southampton scoring system, which assesses factors such as inflammation, discharge, and wound dehiscence. Secondary outcomes included patient-reported measures such as pain (measured on a visual analog scale), time to healing, and the rate of complications, which included infections, hypertrophic scarring, and wound breakdown.

Wound assessments were conducted on postoperative days 1, 7, and 30 by a team of independent evaluators who were blinded to the wound closure method used. Patient satisfaction was gauged using a questionnaire at the 30-day follow-up. Statistical analysis was carried out using SPSS software, with continuous variables compared using t-tests and categorical variables using chi-square tests. A p-value of less than 0.05 was considered statistically significant.

**Figure 1:**
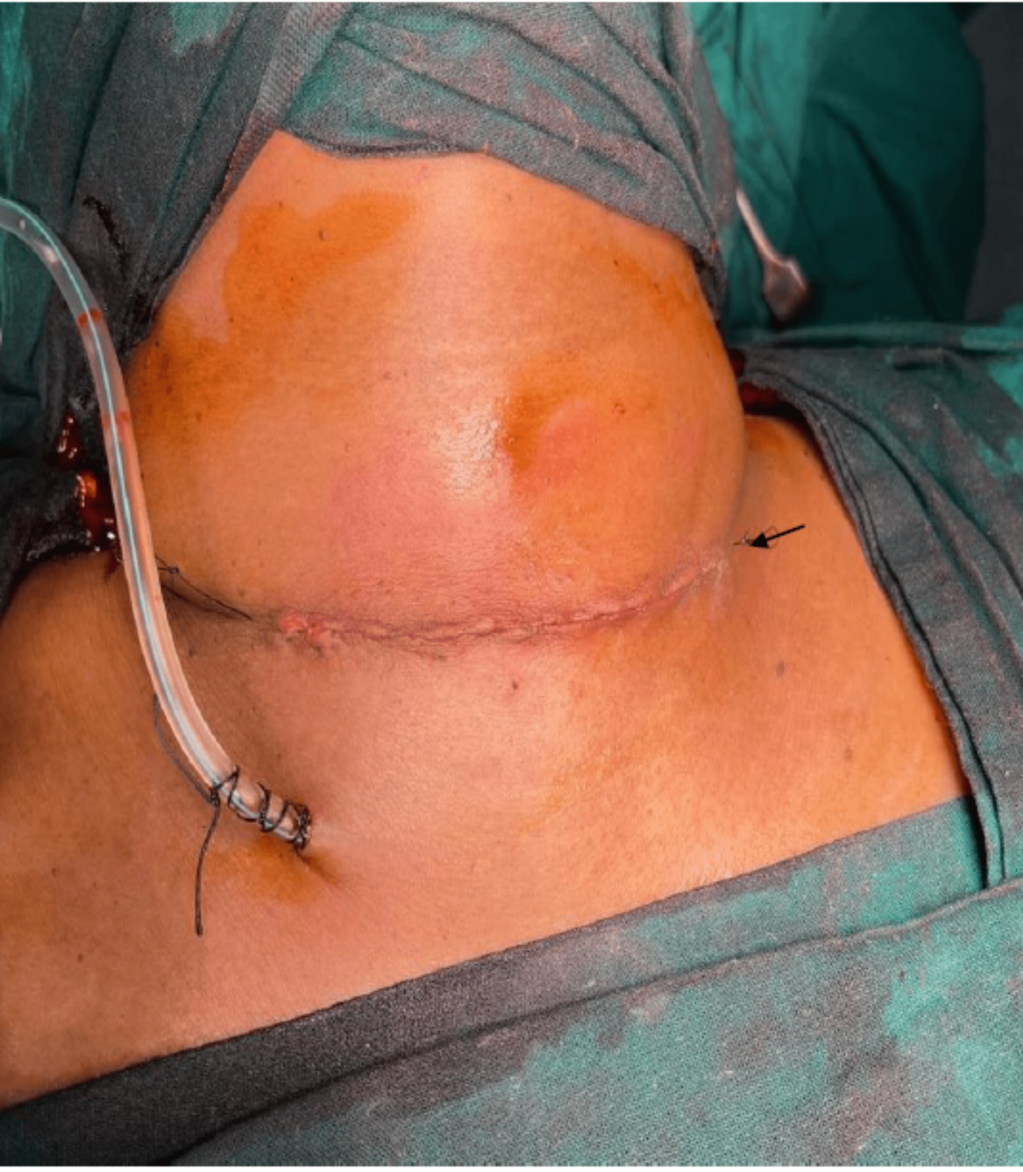
On postoperative day 3, following total thyroidectomy, the skin was closed using glue (black arrow). Original image

A representative image following total thyroidectomy, with the skin closed using glue on postoperative day 3 (Figure 1).

**Figure 2:**
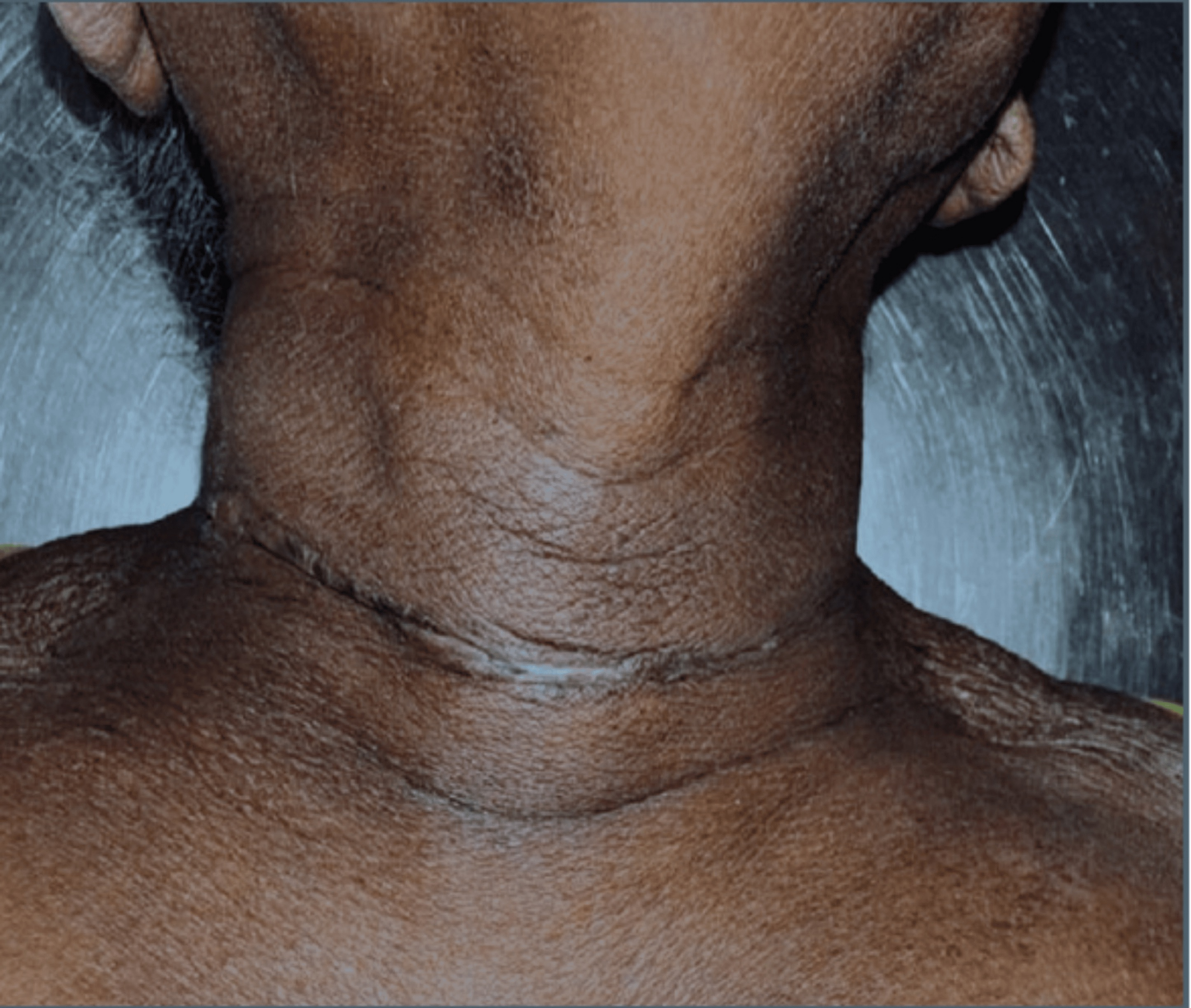
On postoperative day 30, following total thyroidectomy, the skin was closed using cyanoacrylate glue. Original image

A representative image following total thyroidectomy, with the skin closed using cyanoacrylate glue on postoperative day 30 (Figure 2). 

## Results

**Figure 3:**
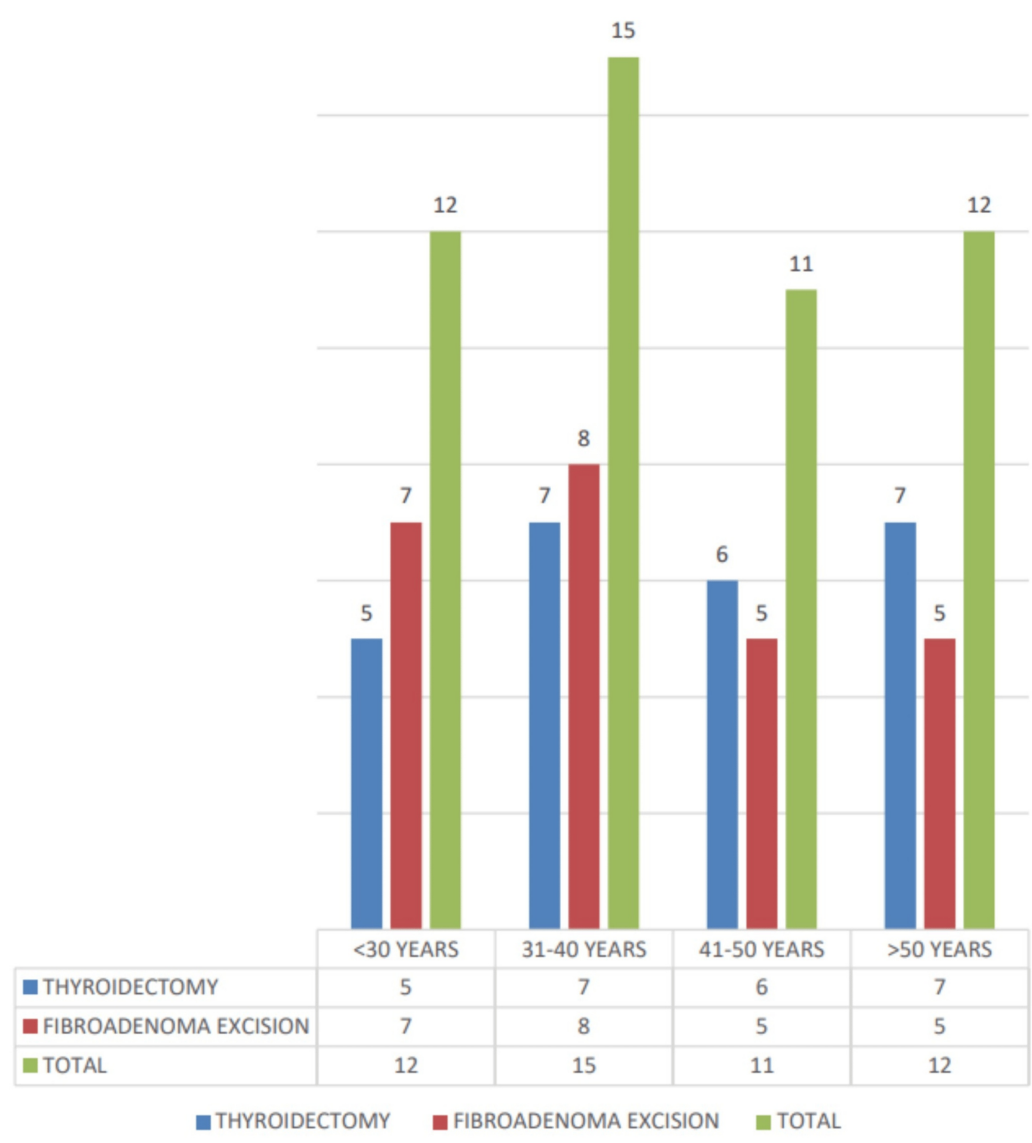
Age distribution.

The sex distribution for thyroidectomy cases shows that 4 males (16.0%) and 21 females (84.0%) underwent the procedure, while for fibroadenoma excision cases, all 25 patients (100.0%) were female, with no male patients. The total number of cases for both procedures was 25 (100.0%) in each group (Figure 3).

**Figure 4:**
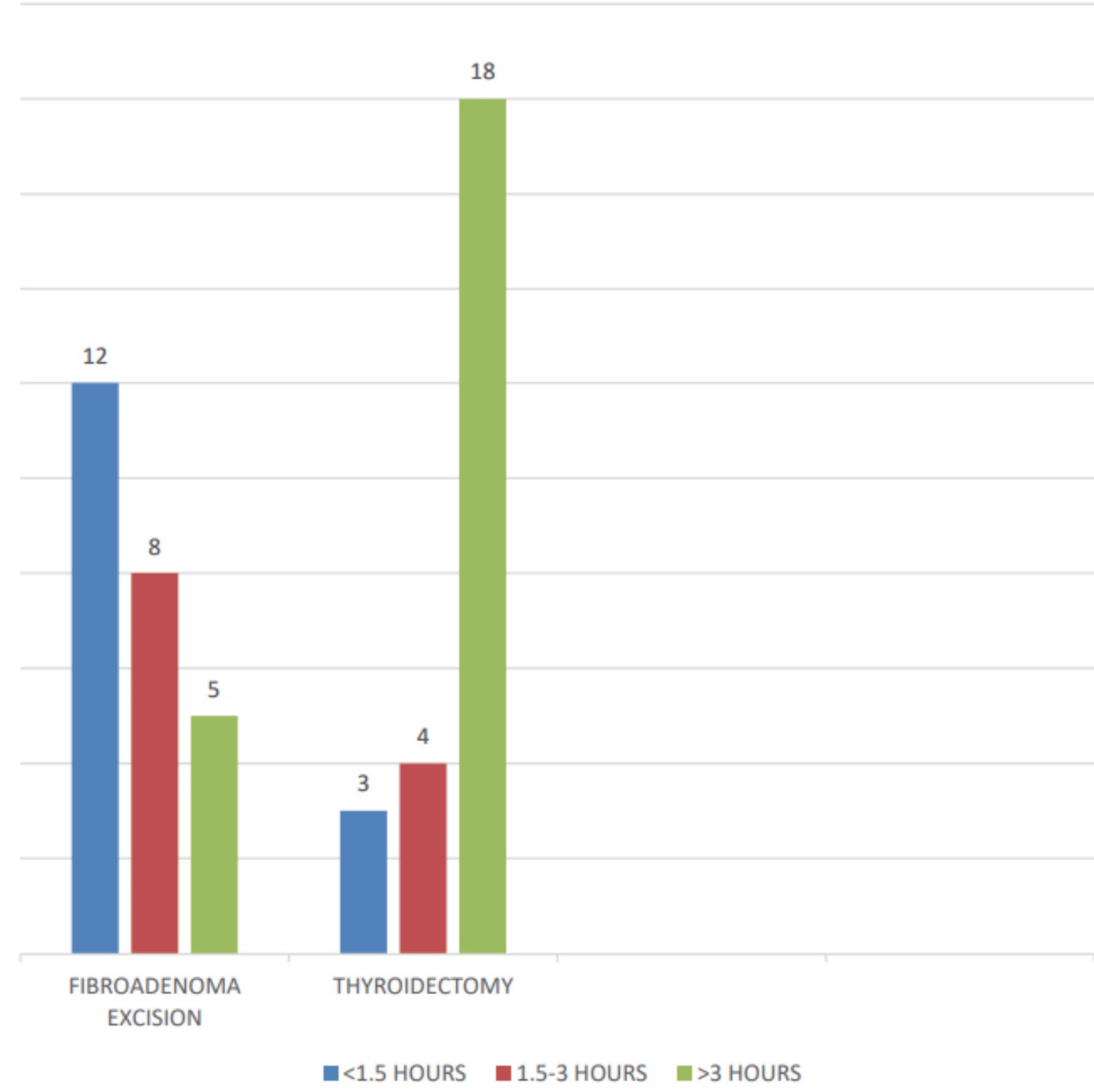
Distribution of duration of surgery.

The duration of surgery for thyroidectomy cases shows that 3 patients (12.0%) had surgery lasting less than 1.5 hours, 4 patients (16.0%) had surgery lasting between 1.5 and 3 hours, and 18 patients (72.0%) had surgery lasting more than 3 hours. For fibroadenoma excision, 12 patients (48.0%) had surgery lasting less than 1.5 hours, 8 patients (32.0%) had surgery lasting between 1.5 and 3 hours, and 5 patients (20.0%) had surgery lasting more than 3 hours. The total number of cases for both procedures was 25 (100.0%) each. The mean duration of surgery for thyroidectomy was 7.56 ± 1.57 hours, while for fibroadenoma excision, it was 1.35 ± 2.4 hours (Figure 4).

**Figure 5:**
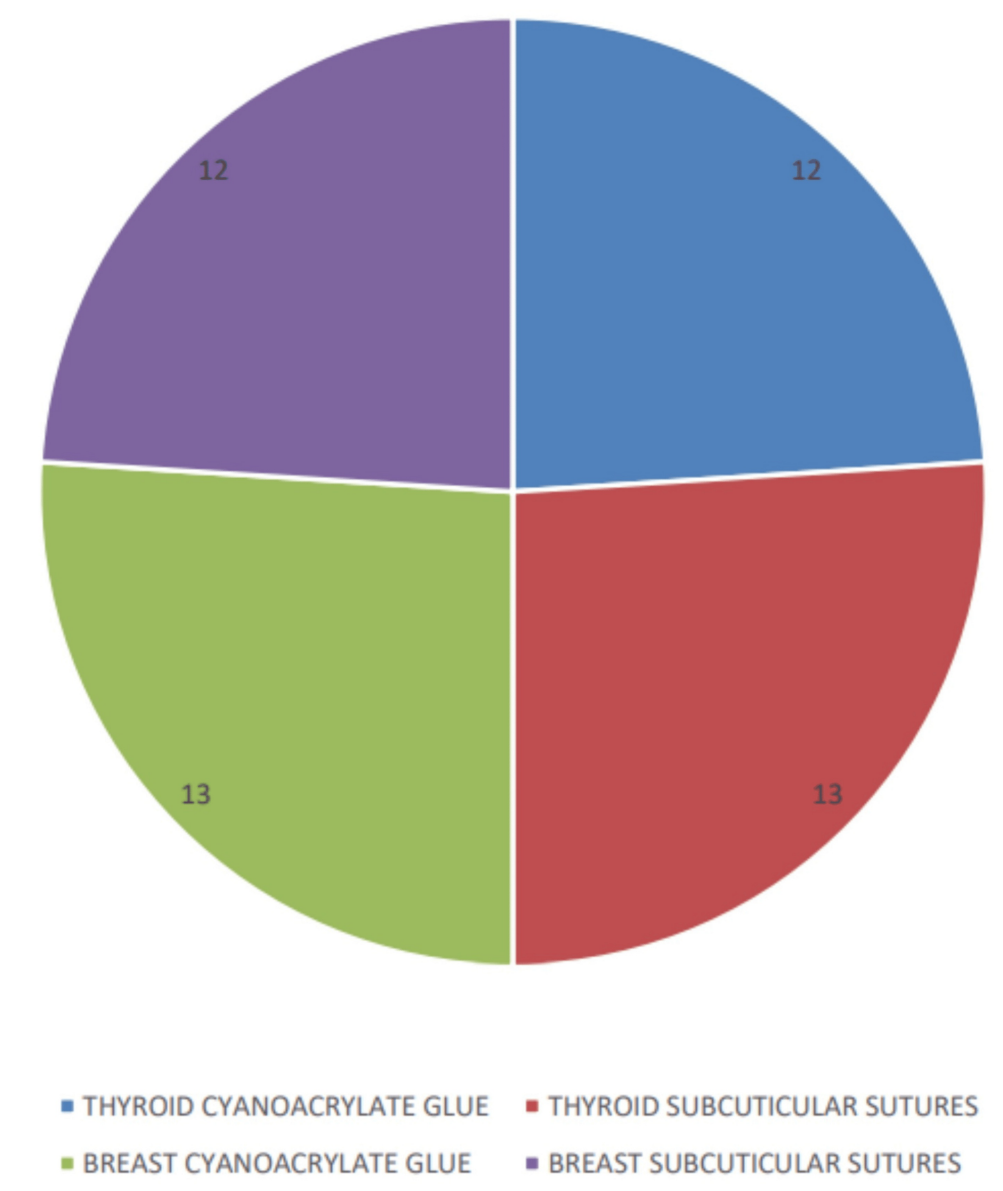
Distribution by wound closure method.

The wound closure method for thyroidectomy cases shows that 12 patients (48.0%) had cyanoacrylate glue used for wound closure, while 13 patients (52.0%) had subcuticular sutures. For fibroadenoma excision cases, 13 patients (52.0%) had cyanoacrylate glue used, and 12 patients (48.0%) had subcuticular sutures. The total number of cases for both procedures was 25 (100.0%) each (Figure 5).

On postoperative day 3, surgical site infections were observed in patients using cyanoacrylate glue and subcuticular sutures. For those with cyanoacrylate glue, 3 patients (12.0%) had a Grade 1a infection, 2 patients (8.0%) had a Grade 1c infection, and no patients (0.0%) had a Grade 2b infection. In the subcuticular sutures group, 7 patients (28.0%) had a Grade 1a infection, 4 patients (16.0%) had a Grade 1c infection, and 3 patients (12.0%) had a Grade 2b infection. The total number of cases for each group was 25 (100.0%), and the p-value was 0.779 (Table 1).

**Table 1:** Comparison of the distribution of surgical site infections on postoperative day 3 using the Southampton scoring system.

Surgical site infection on postoperative day 3	Cyanoacrylate glue n (%)	Subcuticular sutures n (%)	p-value
Grade 1a	3 (12.0)	7 (28.0)	0.779
Grade 1c	2 (8.0)	4 (16.0)	0.779
Grade 2b	0 (0.0)	3 (12.0)	0.779
Total	25 (100.0)	25 (100.0)	0.779

**Figure 6:**
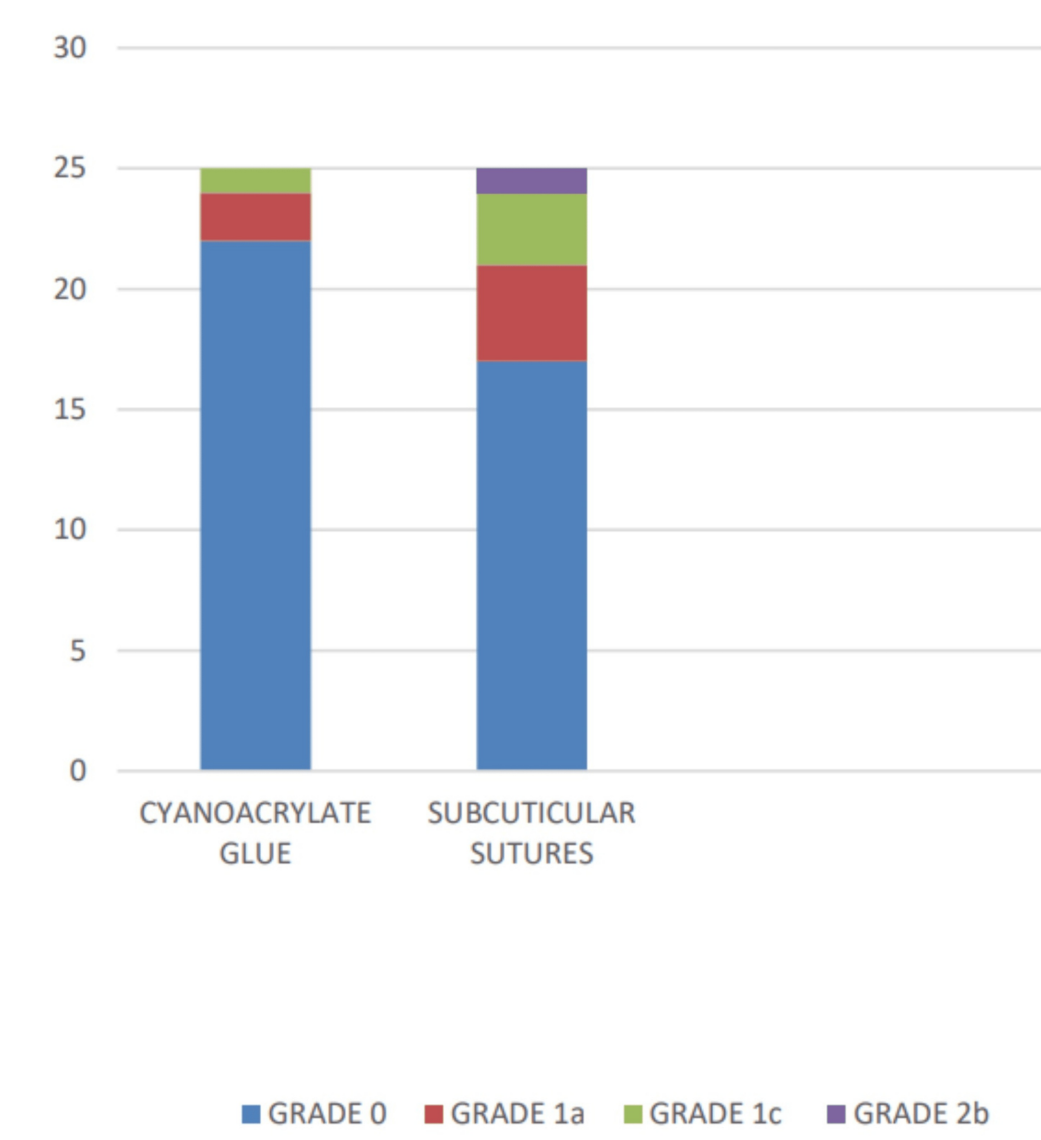
Comparison of the distribution of surgical site infections on postoperative day 7 using the Southampton scoring system.

On postoperative day 7, the comparison of surgical site infections using the Southampton scoring system showed that in the cyanoacrylate glue group, 2 patients (8.0%) had a Grade 1a infection, 1 patient (4.0%) had a Grade 1c infection, and no patients (0.0%) had a Grade 2b infection. In the subcuticular sutures group, 4 patients (16.0%) had a Grade 1a infection, 3 patients (12.0%) had a Grade 1c infection, and 1 patient (4.0%) had a Grade 2b infection. The total number of cases for both groups was 25 (100.0%) each, and the p-value was 0.000 (Figure 6).

**Figure 7:**
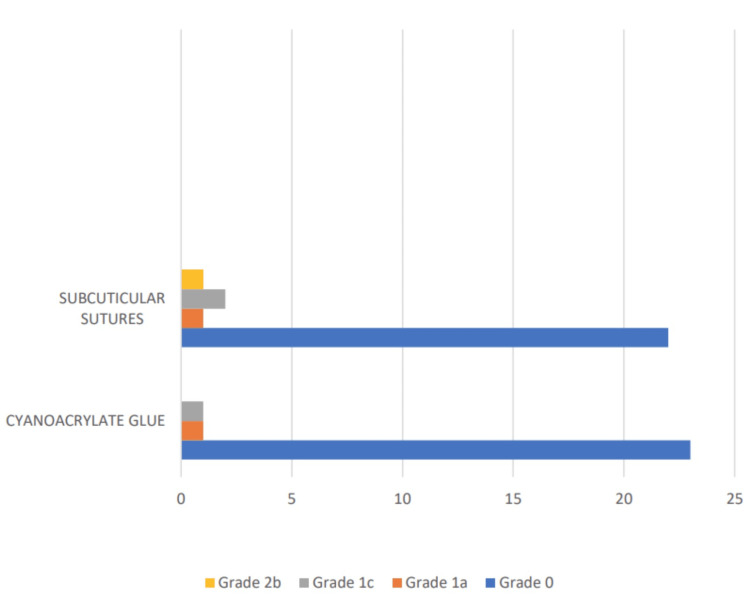
Comparison of the distribution of surgical site infections on postoperative day 30 using the Southampton scoring system.

On postoperative day 30, the comparison of surgical site infections using the Southampton scoring system showed that in the cyanoacrylate glue group, 1 patient (4.0%) had a Grade 1a infection, 1 patient (4.0%) had a Grade 1c infection, and no patients (0.0%) had a Grade 2b infection. In the subcuticular sutures group, 1 patient (4.0%) had a Grade 1a infection, 2 patients (8.0%) had a Grade 1c infection, and 1 patient (4.0%) had a Grade 2b infection. The total number of cases for both groups was 25 (100.0%) each (Figure 7).

## Discussion

This study demonstrates the differences in clinical outcomes between cyanoacrylate glue and subcuticular sutures for wound closure in cosmetic procedures. Both methods are widely accepted, with cyanoacrylate glue serving as a non-invasive option compared to traditional suturing [6,7]. The findings reveal that while both techniques are effective, there are noticeable variations in terms of wound healing, infection rates, and patient satisfaction, as evaluated using the Southampton Scoring System.

One notable observation in the study is the difference in infection rates between the two groups. Cyanoacrylate glue exhibited a lower rate of early postoperative infections than subcuticular sutures, although it was not without complications [8]. The lower infection rate with cyanoacrylate glue may be due to its ability to form a protective barrier over the wound, thereby reducing the risk of bacterial contamination. Conversely, subcuticular sutures, while providing effective closure, could promote bacterial growth in certain cases, especially if wound care is inadequate.

The study also found significant differences in the duration of surgery between the two groups, with cyanoacrylate glue reducing operative time compared to subcuticular sutures [9,10]. This reduction in surgery time can be especially beneficial in busy surgical settings, where efficiency is essential. Additionally, shorter procedures may reduce overall stress on the patient and potentially contribute to faster postoperative recovery.

Patient satisfaction, which plays a key role in cosmetic surgery, was reported to be higher in the cyanoacrylate glue group. Patients in this group experienced less postoperative pain and discomfort, likely due to the less invasive nature of the adhesive method [11,12]. The absence of suture removal, which can cause anxiety or discomfort, may have also contributed to higher satisfaction scores. However, it is important to acknowledge that patient satisfaction can be subjective and influenced by factors such as individual pain thresholds and pre-surgical expectations.

While this study provides valuable insights into the comparative efficacy of cyanoacrylate glue and subcuticular sutures in cosmetic procedures, it is limited by the absence of a standardized postoperative pain scoring system. Although patient-reported pain was qualitatively observed, the lack of objective pain measurement tools, such as a visual analog scale or numerical rating scale, may reduce the reliability and comparability of pain outcomes.

While cyanoacrylate glue offers several advantages, it is not without its limitations. It may not be ideal for all wound types, particularly in areas of high tension or where significant movement occurs [12,13]. In these instances, subcuticular sutures may offer superior strength and provide a more secure closure, reducing the risk of wound dehiscence. Thus, the choice of closure method should be individualized based on both the patient’s condition and surgical requirements.

When considering long-term cosmetic outcomes, both cyanoacrylate glue and subcuticular sutures performed similarly, with no significant differences in scar appearance [14,15]. However, it is important to conduct long-term follow-up studies to determine if these results persist over time, as scar maturation can take months or even years to fully manifest. Changes in patient satisfaction with the aesthetic outcomes may occur as the scar evolves over time.

In this study, both cyanoacrylate glue and subcuticular sutures are effective wound closure methods in cosmetic surgery, each with distinct advantages and drawbacks. Cyanoacrylate glue offers a quicker, less invasive application with higher early patient satisfaction, while subcuticular sutures provide a more durable closure for wounds under tension. Surgeons should consider these factors when deciding on the most appropriate method for each patient, aiming to balance clinical outcomes with patient comfort and satisfaction.

## Conclusions

In conclusion, this study highlights the comparative outcomes of cyanoacrylate glue and subcuticular sutures in cosmetic procedures, emphasizing their respective advantages and limitations. Cyanoacrylate glue offers faster application, reduced surgery times, and higher patient satisfaction in the immediate postoperative period, making it a preferred choice for procedures requiring minimal invasiveness. However, subcuticular sutures provide stronger and more durable wound closure, especially in areas under higher tension. Both methods performed equally well in terms of long-term cosmetic outcomes, with no significant differences in scar appearance. Therefore, the selection of the wound closure technique should be tailored to the specific needs of each patient, taking into account the nature of the procedure and individual patient factors to optimize clinical outcomes and patient satisfaction.
